# Feasibility and Acceptability of the Informant AD8 for Cognitive Screening in Primary Healthcare: A Pilot Study

**DOI:** 10.1155/2014/302834

**Published:** 2014-12-07

**Authors:** YanHong Dong, Tuck Seng Cheng, Keith Yu Kei Tsou, Qun Lin Chan, Christopher Li-Hsian Chen

**Affiliations:** ^1^Department of Pharmacology, National University Health System, Yong Loo Lin School of Medicine, Clinical Research Centre, MD11, Level 5, No. 05-9, 10 Medical Drive, Singapore 117597; ^2^Centre for Healthy Brain Ageing and Dementia Collaborative Research Centre, UNSW Medicine, The University of New South Wales, Level 3, AGSM Building, Sydney, NSW 2052, Australia; ^3^NHG Polyclinics, National Healthcare Group, 6 Commonwealth Lane, Level 7, 01/02 GMTI Building, Singapore 149547

## Abstract

*Objectives.* The utility of informant AD8 for case finding of cognitive impairment at primary healthcare settings is unknown and therefore its feasibility and acceptability for targeted screening at a primary healthcare clinic should be investigated. *Methods.* The informants of older adult patients attending a primary healthcare clinic in Singapore were administered the AD8. Positive screening findings were provided to patients' primary care physicians for referrals to specialist memory clinics. The acceptability of AD8 was evaluated by collecting feedbacks from the informants and primary care physicians. *Results.* 205 patients and their informants were recruited. However, 6 (2.9%) informants were uncontactable, while the majority of the remaining 199 patients with completed AD8 (96.5%, *n* = 192) found it acceptable where 59 (29.6%) patients were deemed cognitively impaired (AD8 ≥ 2). Clinicians (100%, *n* = 5) found the AD8 helpful in facilitating referrals to memory clinics. However, most referral recommendations (81.4%, *n* = 48) were declined by patients and/or informant due to limited insight of implications of cognitive impairment. *Conclusions.* The AD8 can be easily administered and is well tolerated. It detected cognitive impairment in one-third of older adult patients and therefore may be useful for case finding of cognitive impairment in the primary healthcare.

## 1. Introduction

Dementia is a global public health priority [1] with increasing prevalence due to aging populations. The number of people aged 60 and older living with dementia has been estimated to be 24.3 million and is expected to double every 20 years by 2040 [2]. However, rates of unrecognized dementia in the primary healthcare setting were reported to range from 3.2% to 12% in 2003 [3] and have been found to be alarmingly higher at 81.3% in older adults (≥65 years old) residing in Indianapolis [4]. In that study, the authors concluded that most primary healthcare practices were ill prepared to provide dementia screening and diagnosis program. Whilst the prevalence of dementia in community-dwelling Singaporean older adults (≥50 years old) was reported to be 1.3% [5], the rate of unrecognized dementia in the primary healthcare setting in Singapore is unknown and requires investigation. Hence, it is important to establish an effective brief instrument to detect cognitive impairment in older adult patients in a primary healthcare setting.

Several brief cognitive instruments have been suggested for routine screening in primary care [6]. These included performance based instruments (Mini-Cog, the Memory Impairment Screen and General Practitioner Assessment of Cognition (GPCOG)) and informant based instruments (short Informant Questionnaire on Cognitive Decline in the Elderly (IQCODE), GPCOG, and the informant AD8). These instruments were recommended due to short administration time (<5 minutes), ease in administration which does not require highly trained personnel, and good psychometric properties, as well as validation in a primary care or community setting. Of these instruments, the AD8 [7], a recently developed brief “Eight-item Interview to Differentiate Aging and Dementia” cognitive instrument, may be more suitable for case finding of cognitive impairment in Singapore primary healthcare clinics. It has demonstrated good discrimination for uncertain or questionable dementia [7] and consists of 8 questions which track the intraindividual functional decline attributed to cognitive deficits and is therefore less influenced by culture, education, gender, and age [8]. The AD8 follows a yes-no format with the total score based on “yes” response only which can be completed within approximately 3 minutes [7]. A previous study has proposed that the AD8 can be administered to either informants or patients in person or over the phone [9, 10]. However, our recent study at a memory clinic has found that the informant AD8 is superior to the patient AD8, and equivalent to the Minimental State Examination and the Montreal Cognitive Assessment in screening for mild cognitive impairment and dementia in memory clinic patients [11]. However, the utility of the informant AD8 for case finding of cognitive impairment in a primary healthcare setting has not been examined.

Therefore, in this pilot study, we aimed to examine the feasibility and acceptability of the informant AD8 for case finding of cognitive impairment in a primary healthcare setting in Singapore. We hypothesize that (1) the informant AD8 can be efficiently administered to informants of older adult patients aged 60 and above; (2) the informant AD8 can be easily completed in a busy primary healthcare setting; (3) the informant AD8 is clinically relevant at the primary healthcare setting because it can facilitate referrals to a specialist memory clinic.

## 2. Methods

### 2.1. Participants

The study was conducted at the “Chronic Illness” clinic at Bukit Batok Polyclinic in Singapore between March 2012 and April 2012. Eligible patients are those who (1) were aged 60 and above, (2) provided consent, (3) and had an informant with sufficient language skills in English, Chinese, or Malay to complete the AD8. Reasons for declining participation were collected. Informants of recruited patients completed the AD8 independently or with assistance from a trained research psychologist at the waiting area of the clinic. The AD8 was also conducted over the phone for informants who were not present in the clinic. The time taken for test administration and demographic characteristics of patients and informants were collected. This study was approved by the local ethics committee and conducted in conformity with the Declaration of Helsinki. Written informed consent was obtained from all patients and their informants.

The AD8 is an eight-item questionnaire covering judgment, memory, and function. It was developed as an informant-rated measure to track intraindividual functional decline over the past several years attributed to cognitive impairment. It follows a yes-no format (“Yes, A change,” “No, No change,” and “N/A, do not know”) and takes approximately 3 minutes to complete [7]. The total score is a sum of the number of items with a response “Yes, A change.” The AD8 scores of 0-1 can be interpreted as normal cognition, while scores of ≥2 indicate impairment in cognition. A cutoff of 2 was selected according to recent studies in Singapore and a previous community-based study [10, 11]. Patients with the AD8 scores ≥2 were deemed to have cognitive impairment and thus classified as screen positive. The AD8 results of screen positive patients were provided to their attending primary care physicians for consideration of referral to a specialist memory clinic. The referral rate as well as reasons for declining referrals was recorded. The follow-up rate in the specialist memory clinic was tracked. Additionally, feedback of informants and primary care physicians on the informant AD8 was collected to determine acceptability and user-friendliness of this instrument.

### 2.2. Statistical Analyses

All statistical analyses were conducted using Statistical Package for the Social Sciences, Version 20.0 (SPSS Inc., Chicago, IL, USA). Descriptive statistical analyses were conducted to examine the acceptability of the informant AD8. The average time of test administration was calculated. Between-group comparisons were performed using independent-sample *t*-tests for quantitative variables and bivariate analysis including Pearson chi-square tests for categorical variables. Multivariable logistic regression analyses were computed to test the association of all characteristics between groups. Analyses are considered significant when *P* < 0.05.

## 3. Results

**Figure 1:**
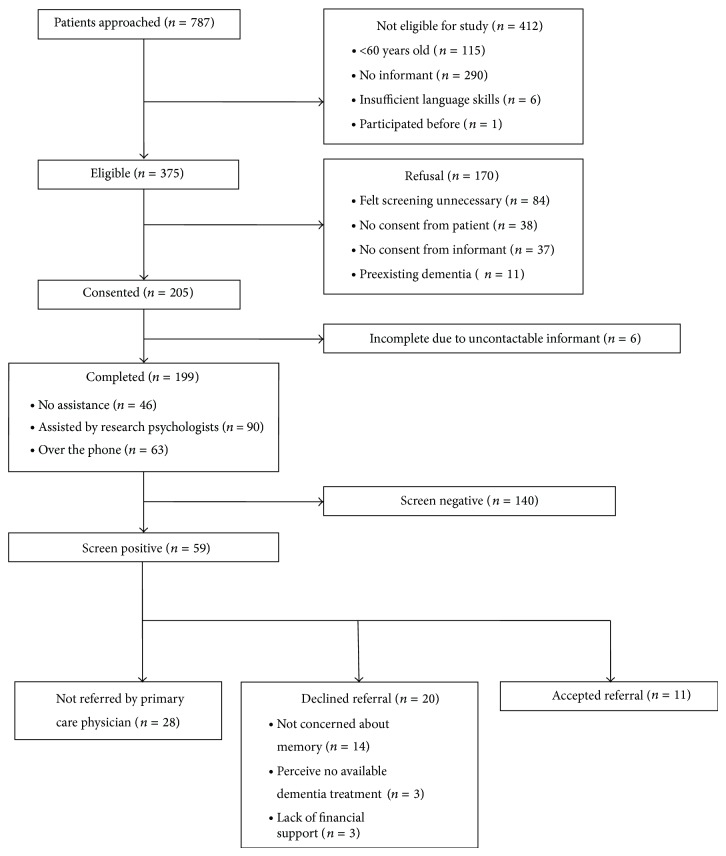
Study inclusion flow chart.

The flow diagram of participation is shown in Figure 1. Approximately half of 787 patients (*n* = 375, 47.6%) attending the “Chronic Illnesses” clinic at the Bukit Batok Polyclinic were eligible for this study. Of those who were eligible, the majority were Chinese (*n* = 309, 82.4%) and female (*n* = 218, 58.1%) with a mean age of 71.4 ± 8.1 years. Major reasons for exclusion, in order of frequency, were (1) no identified informant (*n* = 290, 70.4%), (2) age younger than 60 years (*n* = 115, 27.9%), and (3) others (e.g., insufficient language skills or participated in this study before; *n* = 7, 1.7%). More than half of the eligible patients were accompanied by their informants (*n* = 216, 57.6%) and provided consent for participation (*n* = 205, 54.7%). The reasons for declining participation were (1) patients felt that screening for cognitive impairment was unnecessary (*n* = 84, 49.4%); (2) no consent was provided from either patient (*n* = 38, 22.4%) or their informants (*n* = 37, 21.8%); (3) patients were with a known diagnosis of dementia (*n* = 11, 6.5%).

The study participants' characteristics are shown in Table 1. The participants had a mean age of 72.1 ± 8.7 years with low level of education of 5.8 ± 5.1 years. Most of these patients have multiple medical conditions (2.2 ± 1.1) with high frequency of vascular risk factors such as hypertension (75.9%), hyperlipidemia (74.7%), diabetes (44.7%), and stroke (12.1%). The majority were Chinese (*n* = 166, 81.0%), females (*n* = 123, 60%), and accompanied by informants to the clinic (*n* = 136, 66.3%), and were living with their family members (*n* = 92, 44.9%).

The informants' characteristics are indicated in Table 2. Of the entire recruited sample, 199 (97.1%) informants with a mean age of 52.9 ± 13.9 years and a mean of 10.5 ± 4.2 years of formal education completed the AD8 with a mean time taken of 2.7 ± 1.4 minutes. The remaining 6 (2.9%) informant AD8 were incomplete due to uncontactable informants. Most of the informants were females (*n* = 136, 68.3%) and Chinese (*n* = 158, 79.4%) and were living with participants (*n* = 161, 80.9%) with very frequent contact (*n* = 161, 80.9%). Among the informants, 108 (54.3%) were adult children of the patients, 72 (36.2%) were patients' spouses, 19 (9.5%) were others (e.g., relatives, close friends, and paid caregivers). The majority of the informants required assistance in completing the AD8 (*n* = 90, 45.2%), while a third of the informants were able to complete it over the phone (*n* = 63, 31.7%), and approximately a quarter of informants could complete it independently (*n* = 46, 23.1%) at a busy primary healthcare clinic. Among informants who were present at the clinic, a small minority (*n* = 7, 5.1%) found it unacceptable to complete the AD8 while waiting with patients for medical consultation.

Approximately one-third of patients were screen positive with the scores of informant AD8 ≥ 2 (*n* = 59, 29.6%). The characteristics of screen positive and screen negative patients are shown in Table 3. Independent-sample *t*-tests showed significant mean differences in years of education (*t*(196) = 2.3, *P* = 0.021), composite number of medical conditions (*t*(197) = −2.2, *P* = 0.027), and the AD8 total scores (*t*(197) = −21.0, *P* < 0.001) between these two groups. The screen positive participants had significantly less education and more medical conditions (particularly stroke prevalence), as well as higher AD8 scores than screen negative patients. Furthermore, relative to the screen negative patients, there were more screen positive patients accompanied by informants to the clinic (*χ* ^2^(1) = 10.43, *P* = 0.001) and these patients had more memory difficulty endorsements by their informants (*χ* ^2^(2) = 56.4, *P* < 0.001). The language of choice for the majority of screen positive participants were Chinese compared to screen negative participants (*χ* ^2^(3) = 15.3, *P* = 0.002). However, ethnicity was not significantly different between these two groups. Logistic regression indicated that presence of patients' informants at clinic (OR = 3.12, 95% CI: 1.12, 8.68, *P* = 0.029) and memory endorsement by their informants (OR = 11.45, 95% CI: 5.17, 25.33, *P* < 0.001) predicted patients who were screen positive.

Of 59 screen positive patients, more than half (*n* = 28, 58.3%) were not referred to a specialist memory clinic by primary care physicians, whilst a substantial number of patients (*n* = 20, 41.7%) declined referral to a specialist memory clinic due to (1) limited insight into the health implications of cognitive impairment in patients and their families (*n* = 14, 29.2%) and (2) others (e.g., perceive no available dementia treatment or lack of financial support; *n* = 6, 12.5%). Only a minority of patients (*n* = 11, 18.6%) accepted the referral recommendation to a specialist memory clinic. Of these, approximately one-third (*n* = 3, 27.3%) actually attended a memory clinic where one patient was diagnosed with cognitive impairment and the other two patients were diagnosed to have dementia.

Although a relatively small number of primary care physicians (*n* = 5) completed the satisfaction survey on the usefulness of the informant AD8, all of these physicians considered the informant AD8 helpful in facilitating a referral to specialist memory clinics.

## 4. Discussion

To the best of our knowledge, this is the first AD8 feasibility study conducted in a primary healthcare setting.

The contributions of this pilot study are threefold. First, we have demonstrated that the informant AD8 can be efficiently administered to informants of older adults (≥60 years old) attending a primary healthcare clinic. Second, the informant AD8 can be easily completed in a busy primary healthcare setting. Third, the informant AD8 is clinically relevant at the primary healthcare setting because it can facilitate referrals to a specialist memory clinic.

Our finding that the informant AD8 is feasible and acceptable to patients and informants in a primary healthcare setting in Singapore, that is, almost all recruited informants (97.1%) were available for screening, concurs with a previous population-based study (98%) [12]. Furthermore, we have shown that less than half of the informants required assistance in completing the AD8, while a third of the informants were able to complete it over the phone, and approximately a quarter of informants could complete it independently at a busy primary healthcare clinic.

Finally, the informant AD8 is clinically relevant at the primary healthcare setting because it can facilitate referrals to a specialist memory clinic. Using a cutoff point validated in our memory clinic and from a community study in the United States (AD8 ≥ 2), approximately a third of primary healthcare clinic patients were screen positive, which is higher than the prevalence in the older adults population (aged ≥ 65 years) attending a primary care setting in the Indianapolis region of the states (29.6% versus 13%) [4]. In comparison with patients who screened negative, those with a positive screen were more likely to attend clinic accompanied by their informants who endorsed more on the changes of patients' memory.

However, the rate of no follow-up clinical evaluation in our screen positive patients was higher than the study in Indianapolis (81.4% versus 52.3%). This is mainly due to nonreferral by primary care physicians and limited insight into the health implications of cognitive impairment by patients and their families. Only a minority of patients (18.6%) agreed to be followed up at a specialist memory clinic. Of these, a third of them attended a memory clinic and received a formal diagnosis of cognitive impairment. Few primary care physicians completed the satisfaction survey on the usefulness of the informant AD8. However, all of them considered it helpful in facilitating a referral to specialist memory clinics.

There are several limitations in the present pilot study. First, we used the established cutoff point of the informant AD8 to define cognitive impairment rather than a formal neuropsychological test and clinical evaluation. Future studies should examine cognitive impairment and dementia defined by a formal neuropsychological battery and clinical diagnostic work-up. Second, the screening was conducted over a short period of time (<1 month) in one primary healthcare clinic which limited its generalizability; future studies may include several primary healthcare clinics and screen over a longer time frame. Third, there were a substantial number of patients excluded from the present study (290 out of 412) due to the lack of informant. These patients may require performance-based brief test for the detection of cognitive impairment. Finally, as this was a pilot study, only a small number of patients (*n* = 11, 18.6%) accepted the referral recommendation to a specialist memory clinic. Additionally, we did not explore the reasons for the lack of referral of the screen positive patients from primary healthcare physicians to a specialist memory clinic and the reasons for patients who accepted the referral but did not attend the specialist memory clinic. Future studies should include a larger sample of patients attending primary healthcare and examine these factors by collecting feedback from primary healthcare physicians and interviewing patients who do not attend the memory clinic.

## 5. Conclusion

The informant AD8 can be easily administered and is well tolerated by patients attending a busy primary healthcare clinic in Singapore. It could detect cognitive impairment in one-third of older adult patients accompanied by their informants who endorse memory changes in patients, therefore could be well suited for case finding of cognitive impairment in the primary healthcare setting.

## Figures and Tables

**Table 1:** Patient characteristics.

	*n* (%)
Mean age (mean ± sd)	72.1 ± 8.7
Years of education (mean ± sd)	5.8 ± 5.1
Gender	
Female	123 (60.0)
Male	82 (40.0)
Ethnicity	
Chinese	166 (81.0)
Malay	24 (11.7)
Indian	12 (5.9)
Others	3 (1.5)
Language	
Chinese	119 (58.0)
English	61 (29.8)
Malay	19 (9.3)
Tamil	6 (2.9)
Living situation	
Spouse and adult children	92 (44.9)
Adult children only	57 (27.8)
Spouse only	36 (17.6)
Alone	12 (5.9)
Others	8 (3.9)
Composite number of medical conditions (mean ± sd)	2.2 ± 1.1
Hypertension	151 (75.9)
Hyperlipidemia	148 (74.7)
Diabetes	89 (44.7)
Stroke	24 (12.1)
Smoking	17 (8.5)
TIA	3 (1.5)
Depression	2 (1.0)
Accompanied by informant	136 (66.3)

**Table 2:** Informant characteristics.

	*n* (%)
Mean age (mean ± sd)	52.9 ± 13.9
Years of education (mean ± sd)	10.5 ± 4.2
Average AD8 administration time (minutes) (mean ± sd)	2.7 ± 1.4
Gender	
Female	136 (68.3)
Male	63 (31.7)
Ethnicity	
Chinese	158 (79.4)
Malay	26 (13.1)
Indian	11 (5.5)
Others	4 (2.0)
Language	
English	116 (58.3)
Mandarin	63 (31.7)
Malay	13 (6.5)
Chinese dialects	7 (3.5)
Mode of AD8 administration	
Self	46 (23.1)
Over the phone	63 (31.7)
Assisted	90 (45.2)
Living with participant	161 (80.9)
Relationship with participant	
Adult children	108 (54.3)
Spouse	72 (36.2)
Others	19 (9.5)
Frequency of contact with participants	
Very frequently (everyday)	161 (80.9)
Frequently (several times per week)	13 (6.5)
Often (once per week)	18 (9.0)
Occasionally (<once per week)	7 (3.5)

**Table 3:** Characteristics of screen positive and screen negative patients.

	Screen positive (*n* = 59)	Screen negative (*n* = 140)	*P* value
Mean age (mean ± sd)	73.8 ± 7.7	71.7 ± 9.0	0.110
Years of education (mean ± sd)	4.5 ± 5.2	6.4 ± 5.0	0.021
Gender (*n*, %)			0.586
Female	37 (62.7)	82 (58.6)	
Male	22 (37.3)	58 (41.4)	
Ethnicity (*n*, %)			0.080
Chinese	50 (84.7)	111 (79.3)	
Malay	9 (15.3)	15 (10.7)	
Indian	0 (0.0)	12 (8.6)	
Others	0 (0.0)	2 (1.4)	
Language (*n*, %)			0.002
Chinese	43 (72.9)	71 (50.7)	
English	8 (13.6)	50 (35.7)	
Malay	8 (13.6)	11 (7.9)	
Tamil	0 (0.0)	8 (5.7)	
Living situation (*n*, %)			0.720
Spouse and adult children	25 (42.4)	65 (46.4)	
Adult children only	15 (25.4)	40 (28.6)	
Spouse only	11 (18.6)	23 (16.4)	
Alone	4 (6.8)	8 (5.7)	
Others	4 (6.8)	4 (2.9)	
Composite number of medical conditions (mean ± sd)	2.4 ± 1.2	2.1 ± 1.1	0.027
Hypertension (*n*, %)	47 (79.7)	104 (74.3)	0.418
Hyperlipidemia (*n*, %)	47 (79.7)	101 (72.1)	0.267
Diabetes (*n*, %)	31 (52.2)	58 (41.4)	0.150
Stroke (*n*, %)	12 (20.3)	12 (8.6)	0.020
Smoking (*n*, %)	5 (8.5)	12 (8.6)	0.982
TIA (*n*, %)	2 (3.4)	1 (0.7)	0.157
Depression (*n*, %)	0 (0.0)	2 (1.4)	0.356
Living with informant (*n*, %)	45 (76.3)	116 (82.9)	0.280
Relationship with informant (*n*, %)			0.333
Adult children	33 (55.9)	75 (53.6)	
Spouse	18 (30.5)	54 (38.6)	
Others	8 (13.6)	11 (7.9)	
Frequency of contact with informant (*n*, %)			0.551
Very frequently (everyday)	46 (78.0)	115 (82.1)	
Frequently (several times per week)	5 (8.5)	8 (5.7)	
Often (once per week)	7 (11.9)	11 (7.9)	
Occasionally (<once per week)	1 (1.7)	6 (4.3)	
Accompanied by informant (*n*, %)	50 (84.7)	86 (61.4)	0.001
AD8 total scores (mean ± sd)	3.9 ± 1.8	0.4 ± 0.5	< 0.001
Memory endorsement by informant (*n*, %)	38 (64.4)	19 (13.6)	< 0.001
